# Influence of immediate and permanent obturators on facial contours: a case series

**DOI:** 10.1186/1757-1626-2-6

**Published:** 2009-01-03

**Authors:** Süha Türkaslan, Timuçin Baykul, M Asım Aydın, M Mustafa Özarslan

**Affiliations:** 1 Department of Prosthodontics, Faculty of Dentistry, Süleyman Demirel University, Isparta, Turkey; 2 Department of Oral & Maxillofacial Surgery, Faculty of Dentistry, Süleyman Demirel University, Isparta, Turkey; 3 Department of Plastic & Reconstructive Surgery, Faculty of Medicine, Süleyman Demirel University, Isparta, Turkey

## Abstract

**Introduction:**

Rehabilitation of patients after surgical removal of carcinomas in facial skeleton is one of the most difficult therapies of the stomatognathic system. Significant deformation of tissues, dysfunctions of the stomatognathic system with concurrent biological imbalance of the oral cavity environment frequently affect the treatment to become arduous. Scars and contraction of the oral crevice may cause serious psychological deficiencies that are another aspect of the treatment schedule.

**Case presentation:**

Three Turkish patients ages 46 (male), 61 (male) and 24 (female) who experienced similar operations were rehabilitated with maxillary obturators. The situations was ideal for patient no 1. Patient no 2 could not receive an immediate obturator and patient no 3 rejected using permanent obturator. The paper describes the advantages of a surgical obturator which is constructed before operation and inserted immediately following partial maxillectomy and expresses long term complications when neglecting the use of definite obturator prosthesis, in the light of three cases.

**Conclusion:**

The primary objective of oral-maxillofacial and plastic surgeons and prosthodontists when treating tumors is to eliminate disease and to improve the quality of life including the facial contours which influences the psychological condition of patient. Neglecting immediate obturator construction may cause serious facial appearance problems due to soft tissue contracture. When permanent obturator is rejected, serious contracture of soft tissues and facial disharmony is inevitable.

## Introduction

Prostodontic rehabilitation of maxillectomies is the preferred treatment in most centers over autogenous tissue reconstructions [1-5]. It reveals satisfactory outcomes with respect to speech, nutrition, and facial appearance when the cooperation of the prostodontist begins before the operation and the long term management of the patient is maintained carefully [3,5-9]. When the patient is referred after the operation, optimum results may not be obtained. Thus, if the patient is lost to follow-up and comes out after a long period without using prosthesis, facial disfigurement may be seen to be so severe that resumption of prosthetic rehabilitation may become impossible [10-14]. The aim of this paper is to describe and illustrate the advantages of a surgical obturator that is constructed before operation and inserted immediately following partial maxillectomy and to express the long term complications of neglected use of prosthesis in the light of three cases.

## Case presentation

The following case reports illustrate the benefits of obturator prostheses by emphasizing the advantages of the obturator that was constructed before operation and inserted immediately following maxillary surgery. Denial of using permanent obturator is also demonstrated.

### Case 1

**Figure 1:**
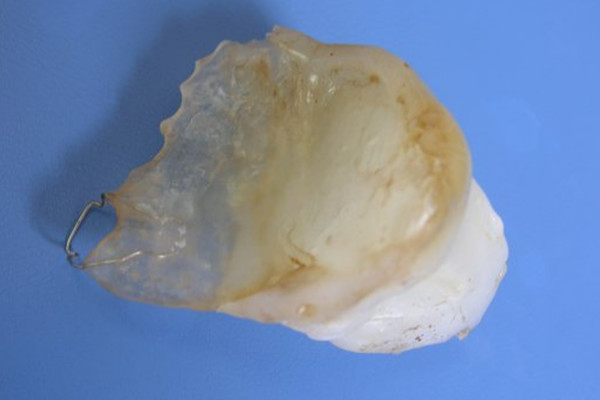
**Immediate obturator with Adams retention clasp**.

**Figure 2:**
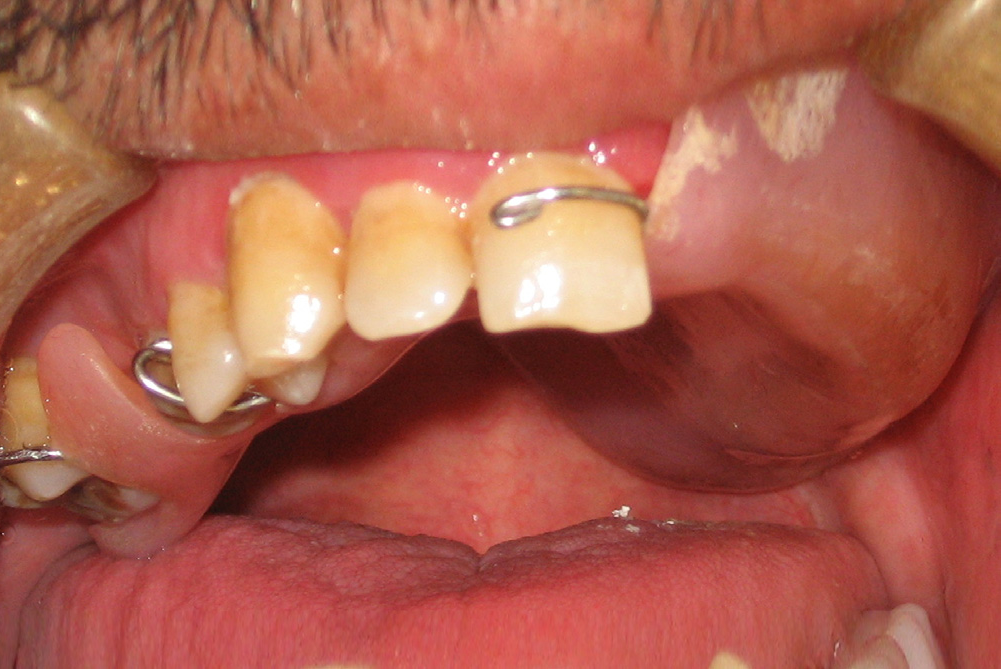
**Intraoral view of interim obturator**.

**Figure 3:**
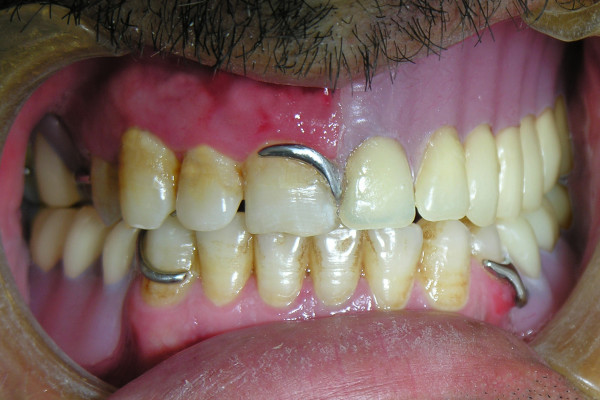
**The permanent obturator in occlusion**.

**Figure 4:**
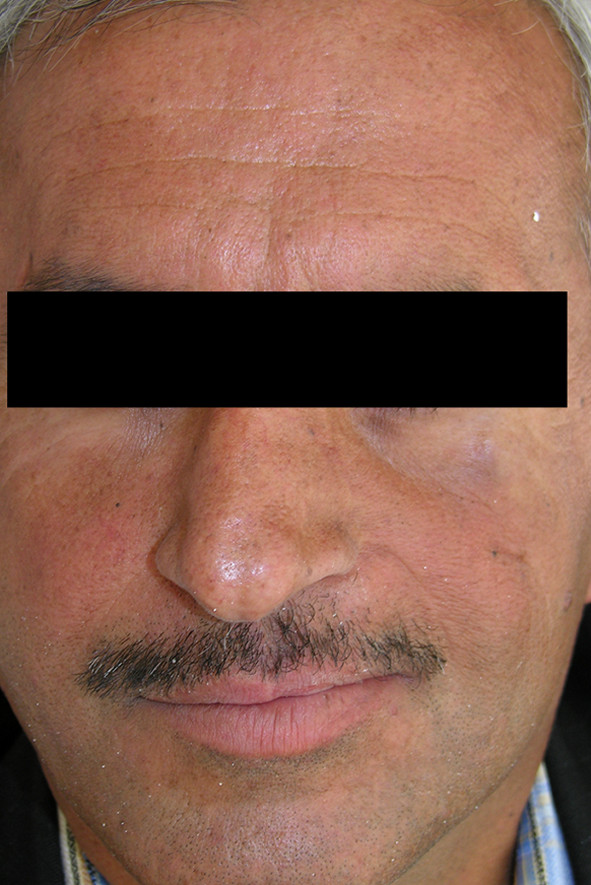
**Extraoral view of patient no 1 who used immediate and interim obturator**.

46-year old male patient presented to his local dentist complaining of pain and swelling associated with his upper teeth. Routine dental diagnostic procedures and periapical radiographs failed to determine the origin of swelling and he was referred to the department of Oral & Maxillofacial Surgery at Suleyman Demirel University, Faculty of Dentistry. A firm swelling was seen to involve on the left side of the maxillae. An incisional biopsy was performed and this revealed the mass to be a Squamous Cell Carcinoma (SCC). The decision following the consultation was to resect the tumor and to obturate the defect with an immediate prosthesis. The patient was informed about the treatment procedure and the immediate obturation which would minimize the alteration of his appearance. Prior to surgery impressions of the maxilla and mandible were obtained and the cast models were attached to a semiadjustable articulator. The predicted excision was performed on the maxillary model. An immediate obturator with 1 cm extension into the resected side was constructed with adams retention clasp on the right second molar teeth in the preserved side. Under general anesthesia the left side of the maxillae was resected together with the lower third of the nasal septum. After the removal of the tumor, tissue conditioning material was placed over the extension of the immediate obturator to fit the surgical defect accurately and to support the defect area and split-skin grafts (Fig. 1). Greater retention and stability was achieved with peridental ligatures. The patient demonstrated good postoperative progress and 10 days after the surgery the immediate prosthesis was subsequently replaced by an interim obturator (Fig. 2) which was then replaced with a definitive prosthesis after three months (Fig. 3). Little soft tissue collapse was observed in the medial part of the zygoma as the region failed to support the lateral extension of the permanent obturator. The problem was tolerated by the patient and he has been satisfied with his appearance (Fig. 4) as well as the functions.

### Case 2

**Figure 5:**
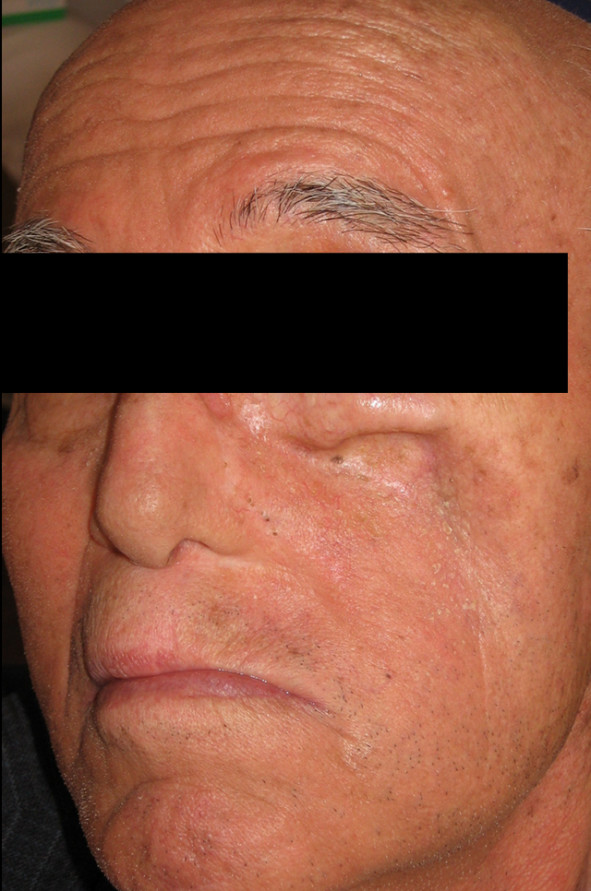
**Extraoral view of patient no 2 who received no immediate obturator, left mid-facial region collapsed**.

61 years old male patient was sent to the department of Maxillofacial Surgery with the suspicion of a malign neoplasm in the left side of maxillae due to causeless mobility of left molar teeth and swelling. The biopsy revealed a Squamous Cell Carcinoma (SCC) and hemimaxillectomy was planned for the patient. The patient preferred to receive the surgical operation and adjuvant radiotherapy in a different city where his children lived. The patient returned for prosthetic rehabilitation after 3 months. The history revealed that he has used neither an immediate nor an interim prosthesis. A definitive prosthesis was constructed following an interim obturator for 3 weeks period. The adversely contracted soft tissues did not permit an ideal prosthetic rehabilitation (Fig. 5).

### Case 3

**Figure 6:**
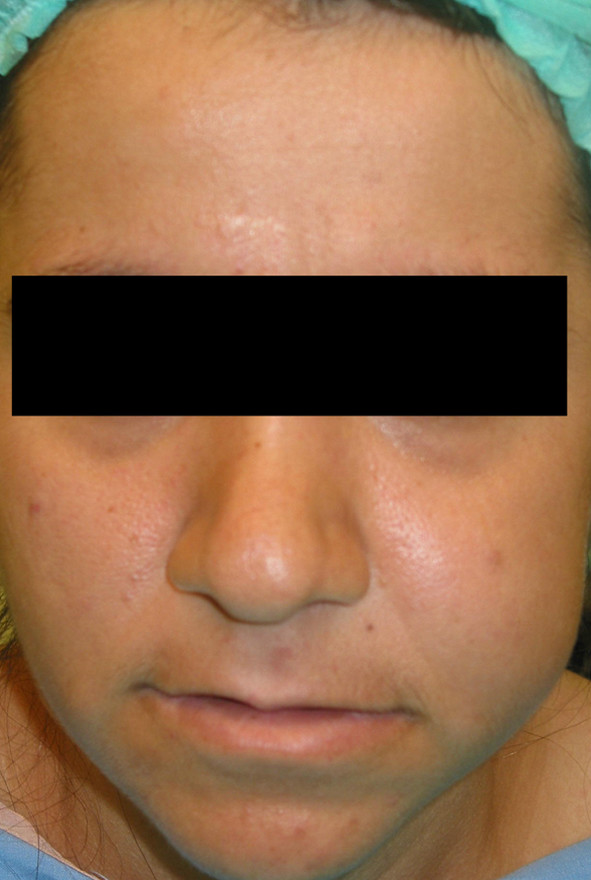
**Pre-op photograph of patient no 3**.

**Figure 7:**
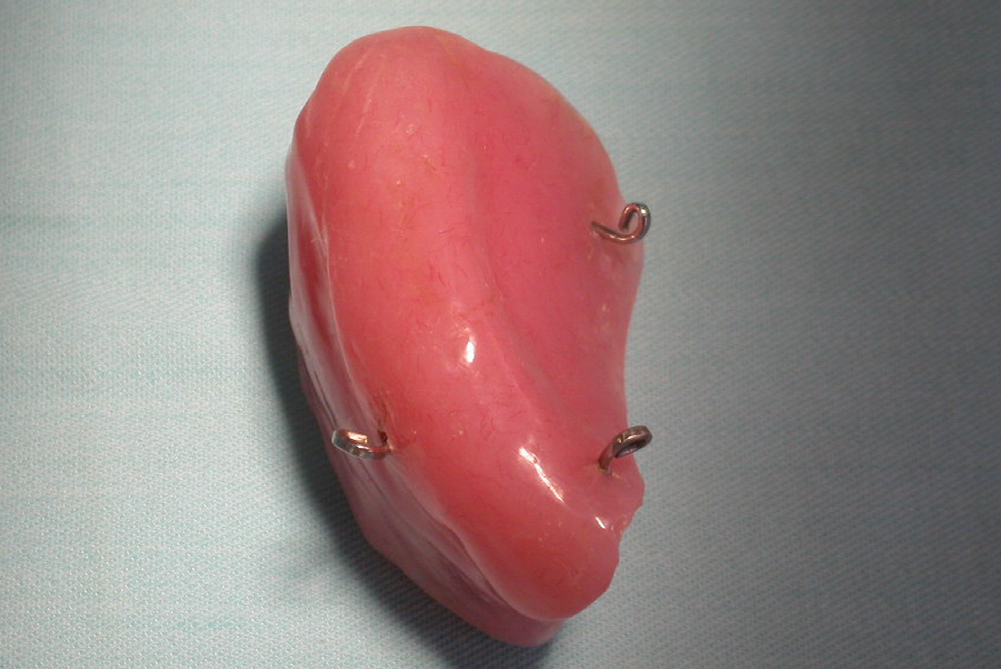
**Spiessl type immediate obturator with loops inserted**.

**Figure 8:**
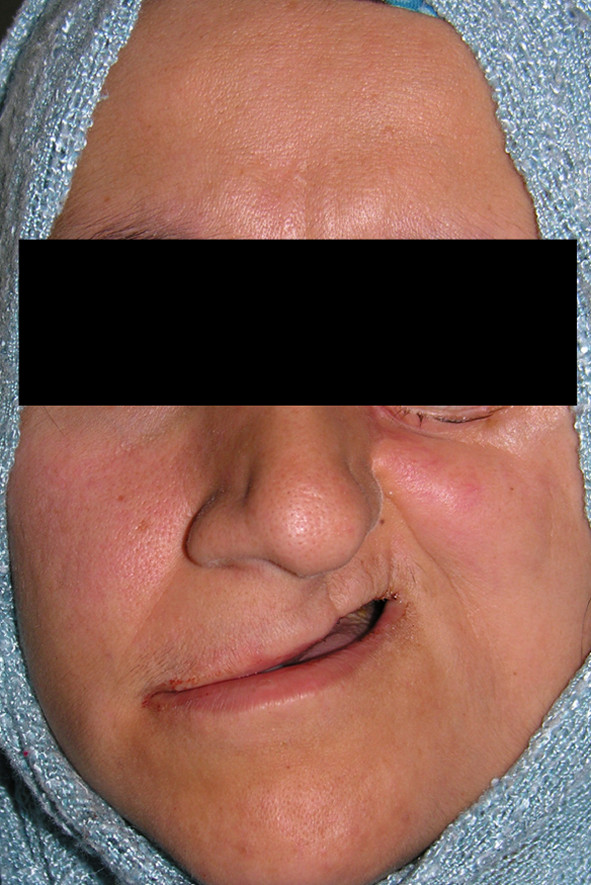
**Facial view of patient no 3 after 5 years surgery**.

24 years old female patient was referred with a swelling localized on her left cheek (Fig. 6). The biopsy revealed an Adenoid Cystic Carcinoma (ACC) and total maxillectomy was performed. Spiessl type surgical obturator was constructed on cast models obtained prior to surgery utilizing polymethyl metacrilate resin material with loops inserted for perizygomatic and transmaxillary ligature (Fig. 7). On completion of surgery the prepared obturator was relined with tissue conditioning material to supply maximum adaptation. The prosthesis was held in position with transmaxillary and circumzygomatic ligatures. No nasogastric tubing was required as oral feeding was immediately feasible. The obturator was removed 10 days postoperatively and first impression was obtained for the model that will be used to fabricate an individual impression tray. Definitive impression was obtained for transitional obturator but she insistently refused to continue prosthetic reconstruction. Many attempts to reach and persuade her for completing the procedure were of no use. A Serious deformity was seen 5 years after surgery (Fig. 8) when she returned requesting some restoration of her facial appearance. As she was found to have lung metastases, neither a surgical procedure nor a prosthetic rehabilitation could be planned.

## Discussion

Neglecting timely prosthodontic cooperation may cause inappropriate facial contour which is almost impossible to reconstruct [3,9,14]. In the reported cases, both patients have experienced similar surgical operations. No complication experienced in patient no 1, but patient no 2 did not have the opportunity to receive immediate obturator where patient no 3 rejected to continue the treatment procedure for the construction of definite obturator. In the absence of immediate obturation, soft tissues remain unsupported and collapse dramatically and aesthetic and/or possible psychological problems may occur [12]. Patient no 3 did not have the chance to use immediate obturator during the construction of the interim obturator since a spiessl type immediate obturator can not be reinserted once removed from the defect area. When she was recalled for definite impression she refused to continue prosthetic reconstruction as she explored the defect with her tongue and by the time she was aware of the extent of the surgery her psychology was negatively affected and many attempts to reach and persuade her for carrying out the procedure was ineffective. The interim obturator was constructed by use of the first cast model and the patient was suggested to use it with tissue conditioning material but all the endeavours were refused by the patient. As immediate obturator, definitive obturator also acts for supporting soft tissues. The impact is more dramatic since the period of time is longer for definitive obturator.

The borders of the defect may collapse more rapidly if support of the immediate obturator is neglected and in a few weeks after surgery – the healing period – especially the anterior and lateral border of the defect migrates towards the center of the defect causing facial aesthetic problems. Supporting soft tissues prevents the continuing of the migration and collapse of the soft tissues. Long term lack of support leads to slower migration but bigger problems which is hard or nearly impossible to treat or reconstruct. For the patient no 3 Levator Anguli Oris and Levator Labi Superior muscles may have been contracted and elevated the comissura as they originate from the maxilla which is already resected. Zygomaticus Major and Minor muscles may also have an effect on the elevation of the comissura considering the surgical procedure. Nevertheless the problem is more than an elevation of the comissura but a collapse of the left mid facial region which even causes the deviation of the nose hip. Therefore lack of support and soft tissue contraction due to radiotherapy is thought to have been more effective on the facial deformity.

Immediate prosthetic replacement is a successful and time-saving procedure that may afford many advantages in the surgical and postoperative management of the patient [4]. Serving as a surgical aid, immediate obturator supports skin grafts with optimum pressure providing their close adaptation to the cavity walls. During the healing phase, the soft tissues are well supported and the contraction of scar tissue is kept at a minimum level [10,13]. Although there is a possible risk of tissue contraction due to radiotherapy immediate obturators may resist rebound of non-supported soft tissues. The resection site is protected from food debris, contaminants and trauma, which may delay healing or dislodge a skin graft. Consequently healing process is provoked and accelerated. After the operation, patients are able to swallow food more readily and to resume a normal diet at an earlier stage[10] which leads to shorter recovery period. The denture component of the obturator allows mastication of soft foods initially and harder foods several days later [3]. Speech is minimally altered and in many instances remains nearly unchanged [7,8]. Exploration of the defect by the tongue is prevented, and the patient remains unaware of the dimensions of the surgery. By maintaining facial contour and aesthetics, patients are psychologically better equipped to face rehabilitation [12]. Experience in wearing an immediate obturator conditions the individual to accept the subsequent prosthetic treatment with equanimity.

## Conclusion

The use of immediate obturators is essential for the optimum rehabilitation of oral functions and cosmetics with prosthetics as well as the maintenance of definitive prosthesis. Immediate obturators support soft tissues after surgery and minimize scar contracture and disfigurement that may have a positive effect on the patients' psychology. Avoiding immediate obturator construction may cause serious facial appearance problems due to soft tissue contracture. When wearing the permanent obturator is neglected, the dynamics of non-supported soft tissues change towards serious contracture and facial disharmony.

## Consent

Written informed consent was obtained from the patients for publication of this case report and accompanying images. A copy of the written consent is available for review by the Editor-in-Chief of this journal.

## Competing interests

The authors declare that they have no competing interests.

## Authors' contributions

TB and MAA operated the patients no 1 & 3. TB also diagnosed the patient no 2. ST and MMO constructed the prosthesis for both three patients and prepared draft manuscript. All authors read and approved the final manuscript.
